# Editorial: The human brain multiscale imaging challenge

**DOI:** 10.3389/fnana.2022.1060405

**Published:** 2022-10-27

**Authors:** Irene Costantini, Markus Axer, Caroline Magnain, Patrick R. Hof

**Affiliations:** ^1^European Laboratory for Non-Linear Spectroscopy, University of Florence, Florence, Italy; ^2^Department of Biology, University of Florence, Florence, Italy; ^3^National Institute of Optics, National Research Council, Sesto Fiorentino, Italy; ^4^Research Centre Jülich, Institute of Neuroscience and Medicine, Jülich, Germany; ^5^Department of Radiology, Athinoula A. Martinos Center for Biomedical Imaging, Massachusetts General Hospital, Boston, MA, United States; ^6^Department of Radiology, Harvard Medical School, Boston, MA, United States; ^7^Nash Family Department of Neuroscience and Friedman Brain Institute, Icahn School of Medicine at Mount Sinai, New York, NY, United States

**Keywords:** human brain, imaging, multiscale, big data, tissue clearing, cellular atlas

## Introduction

The combination of tissue preparation techniques (Costantini et al., 2019), advanced optical microscopy (Abdelfattah et al., 2022), and big data analysis is revolutionizing the way of studying the brain anatomy (Ueda et al., 2020). These techniques already allowed mapping of cell distribution or reconstruction of neuronal circuits in whole mouse brains (BRAIN Initiative Cell Census Network, 2021; Silvestri et al., 2021). However, the analysis of the human brain is still in its infancy. Such specimens present specific challenges that need to be solved in comparison to animal models: massive dimension of the specimen (up to several cm^3^), geometry, variability of post-mortem fixation conditions and storage, presence of blood inside the vasculature, autofluorescence signals from lipofuscin-type pigments, and consistency of cellular labeling. In addition, alteration of antigens, due to fixation and storage conditions, may prevent reliable immunostaining (Weiss et al., 2021; Pesce et al., 2022). Various optical technologies have started to address human brain reconstruction in combination with advanced staining methods or relying on label-free detections (Axer et al., 2016; Wang et al., 2018; Menzel et al., 2020; Costantini et al., 2021a) but much remains to be done. Moreover, the capability of achieving the reconstruction of human brains has also raised the problem of creating new software platforms that enable to manage, analyze, and share TB-sized volumetric images (Tyson and Margrie, 2022). In this collection various methodologies are proposed to perform human brain study from the macro- to the microscale. These studies are summarized in the following paragraphs.

## Comparison of different tissue clearing methods for three-dimensional reconstruction of human brain cellular anatomy using advanced imaging techniques

In this study, Scardigli et al. evaluated different tissue transformation approaches to find the best solution to uniformly clear and label all neurons in the human cerebral cortex to perform 3D reconstruction with light-sheet fluorescence microscopy (LSFM) (Hillman et al., 2019). They applied to adult human brain samples various tissue transformation protocols: CLARITY (Chung et al., 2013; Costantini et al., 2015), SWITCH (Murray et al., 2015; Costantini et al., 2021b), SHIELD (Park et al., 2018), and ExM (Expansion Microscopy) (Chen et al., 2015). Then they optimize a specific procedure to obtain homogeneous staining of the samples with high-density epitopes marker such as NeuN (neuronal nuclear antigen). Finally, they performed mesoscopic high-resolution 3D reconstruction of the successfully cleared and immunostained samples with a custom-made LSFM.

## Scatterometry measurements with scattered light imaging enable new insights into the nerve fiber architecture of the brain

Menzel et al. developed a method based on visual light scattering (Scattered Light Imaging SLI, introduced in Menzel et al., 2021) to infer fiber orientations, particularly in crossing fiber configurations, in unstained histological brain sections. They used a display with individually controllable light-emitting diodes to measure the full distribution of scattered light behind the sample (scattering pattern) for each image pixel recorded at once, enabling scatterometry measurements of whole brain tissue samples. Finally, they performed SLI scatterometry measurements of a human brain section with 3-μm in-plane resolution, demonstrating that the technique is a powerful approach to gain new insights into the nerve fiber architecture of the human brain and to construct a detailed network model of the brain.

## Structural brain imaging predicts individual-level task activation maps using deep learning

In the presented work, Ellis and Aizenberg aim to obtain information that can accurately predict variations in task activation between individuals from structural imaging. To this end, they trained a convolutional neural network to use structural imaging (T1-weighted, T2-weighted, and diffusion tensor imaging) to predict 47 different functional MRI task activation volumes across seven task domains. These results obtained indicate that indeed structural imaging contains information that is predictive of inter-subject variability in task activation mapping and that cortical folding patterns, as well as microstructural features, could be a key component to linking brain structure to brain function.

## Three dimensional-arterial spin labeling evaluation of improved cerebral perfusion after limb remote ischemic preconditioning in a rat model of focal ischemic stroke

The purpose of Zheng et al. is to investigate the application of 3D arterial spin labeling (3D-ASL) (Zhang et al., 2015) for evaluating distal limb ischemic preconditioning (Basalay et al., 2020) to improve acute ischemic stroke (AIS) perfusion. The study is divided in two analysis. The first one is conducted on 40 patients with AIS and 15 healthy individuals in whom diffusion-weighted imaging (DWI), magnetic resonance angiography (MRA), and 3D-ASL, and cerebral infarct volume and cerebral blood flow (CBF) were measured in the area of the infarct lesion. The second part is performed on rats to characterize the cerebral artery occlusion (MCAO) in correlation with limb remote ischemic preconditioning (LRP). From the work on patients the authors found that hypertension and internal carotid atherosclerosis are high-risk factors for ischemic stroke, and CBF values in the infarct area are significantly lower than those in the corresponding regions of the contralateral side. From the second part of the study, they found that LRP reduced cerebral infarct size and improved neurological function in rats after ischemic stroke, while CBF measurements reflected a corresponding improvement in perfusion. The authors concluded that 3D-ASL can be used to evaluate LRP to improve stroke perfusion, and its protective effect may be closely related to LRP-induced vascular regeneration.

## Conclusion

Characterizing the human brain cytoarchitecture, myeloarchitecture, and angioarchitecture, remains one of the biggest challenges of neuroscience. Optical imaging is a powerful tool to analyze such networks and their relationships to cellular anatomy at the microscopic level, which is not achievable by traditional imaging techniques such as dMRI and could lead to better understanding of connectivity, brain development and pathology. Addressing the morphomolecular specificities of cellular diversity globally in the human brain at multiple levels of resolution requires a large-scale, convergent efforts from several disciplines. The papers included in the present collection demonstrates that only the combination of knowledge among various fields, methods, and modalities allows describing the complexity of the brain in an integrated and useful manner. This approach reflects the efforts of massive projects such as the BRAIN Initiative Cell Census Network (BICCN), the BRAIN Initiative Cell Atlas Network (BICAN), and the Human Brain Project (HBP). Realizing human brain atlases with a comprehensive structural and molecular characterization at the cellular level will allow to determine the roles of the different cell types in health and disease, explain the functional alterations occurring in specific cell populations and brain regions as part of a given disease process, opening the possibility of deeply understanding brain's functionalities, while providing accessible, permanent high-quality datasets to the scientific community.

## Author contributions

All authors listed have made a substantial, direct, and intellectual contribution to the work and approved it for publication.

## Funding

This project received funding from the European Union's Horizon 2020 Framework Programme for Research and Innovation under the Specific Grant Agreement No. 785907 (Human Brain Project SGA2) and No. 945539 (Human Brain Project SGA3), from the General Hospital Corporation Center of the National Institutes of Health under award number U01 MH117023, and from the Italian Ministry for Education in the framework of Euro-Bioimaging Italian Node (ESFRI research infrastructure). This project has also been made possible in part by Grant No. 2019-198101 from the Chan-Zuckerberg Initiative DAF, an advised fund of the Silicon Valley Community Foundation. Finally, this research was carried out with the contribution from Fondazione CR Firenze.

## Conflict of interest

The authors declare that the research was conducted in the absence of any commercial or financial relationships that could be construed as a potential conflict of interest.

## Publisher's note

All claims expressed in this article are solely those of the authors and do not necessarily represent those of their affiliated organizations, or those of the publisher, the editors and the reviewers. Any product that may be evaluated in this article, or claim that may be made by its manufacturer, is not guaranteed or endorsed by the publisher.

## Author disclaimer

The content of this work is solely the responsibility of the authors and does not necessarily represent the official views of the National Institutes of Health.
